# A novel approach for quantifying elongated airborne mineral particles (EMPs) using an automated scanning electron microscope (SEM)

**DOI:** 10.1016/j.atmosenv.2025.121217

**Published:** 2025-05-05

**Authors:** Anushka Elangasinghe, Hamesh Patel, Kim N. Dirks, Ayrton Hamilton, Wenxia (Wendy) Fan, Shuoyu Chen, Nick Talbot, Shanon Lim, Jed Januch, Martin Brook, Brett Wells, David E. Williams, Perry Davy, Woodrow Pattinson, Jennifer A. Salmond

**Affiliations:** a School of Environment, Faculty of Science, University of Auckland, Private Bag, 92019, Auckland, New Zealand; b Mote Limited, 40a George Street, Mount Eden, Auckland, New Zealand; c International Laboratory for Air Quality & Health (ILAQH), Queensland University of Technology, 2 George Street, Brisbane, Queensland, 4000, Australia; d Department of Civil and Environment Engineering, Faculty of Engineering, University of Auckland, Auckland, New Zealand; e Environment Southland, Cnr North Rd &, Price Street, Waikiwi, Invercargill, New Zealand; f Pattle Delamore Partners, Level 2/109 Fanshawe Street, Auckland, 1010, New Zealand; g U.S. Environmental Protection Agency, Region 10, 7411 Beach Drive East, 98336, Port Orchard, WA, USA; h School of Chemical Sciences, Faculty of Science, University of Auckland, Private Bag, 92019, Auckland, New Zealand; i Institute of Geological and Nuclear Sciences, Wellington, New Zealand

**Keywords:** Asbestiform fibers, Automated analysis, SEM, Erionite

## Abstract

Exposure to carcinogenic elongated mineral particles (EMPs), such as erionite, found in rocks and released into the air by construction, quarrying, or roading activities, poses a significant possible health risk due to their respirable size and potential for airborne dispersion. The detection of EMPs in the air is typically achieved by filter sampling and subsequent examination using a range of microscopic methods, including phase contrast microscopy (PCM) and scanning electron microscope (SEM). Such analyzes require the manual searching for fibers through many image fields and are both labor-intensive and time-consuming. Moreover, these methods do not result in conclusive particle identification, limiting their effectiveness in large-scale monitoring programmes. This paper introduces a novel methodology for the automated detection and quantification of EMPs using an automated SEM with energy dispersive spectroscopy (EDS) to identify fibers on pre-sampled polycarbonate (PC) filters. This method provides a streamlined workflow for fiber identification based on their size, morphology, and elemental composition. Performance evaluation (PE) standards were prepared by spiking filters with a series of known concentrations of one EMP, namely erionite, and fiber concentrations were measured using the automated SEM-EDS approach. Our results demonstrate a linear relationship (R^2^ = 0.98***) between the erionite mass percentage in a bulk sample and the fiber counts in an aerosolized air volume, with a detection limit of 7.4 f/cc. The approach can be optimized based on the time available for analysis and the choice of detection limit suitable for the specific site and application. Additionally, the automated SEM-EDS method has been applied to real-world air samples collected from Auckland, New Zealand, showing promising results for fiber detection in complex environmental matrices.

## Graphical Abstract

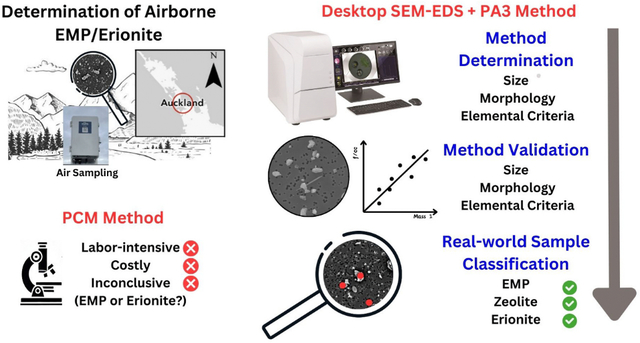

## 1. Introduction

Microfibers refer to natural or synthetic fibrous materials with a thread-like structure (Cao et al., 2024). According to the World Health Organization (WHO), inhalable fibers may pose risks to human health and are defined as particles longer than 5 μm with a width less than 3 μm and a length-to-width ratio greater than 3:1 (WHO, 1986). Hazardous fibers are typically small, durable, and sufficiently fibrous for them to be considered a cancer risk as well as contributing to other adverse health effects (Beaucham et al., 2018).

Microfibers may be abundant in ambient air and are composed of a diverse range of materials, including mineral, microplastics and organic materials. Some fly ash particles may also form fiber-like aggregates (Cao et al., 2024). The toxicological properties and health impacts of microfibers vary depending on their physicochemical characteristics and origin. Research indicates that fibrous particles in the atmosphere may exhibit greater toxicity compared to other particulate morphologies (Dong et al., 2006). Accurate collection and characterization of airborne microfibers, the establishment of standardized analytical protocols, and a comprehensive understanding of their health effects are essential for informing public health policies and developing effective mitigation strategies. Among these microfibers found in the ambient air, are elongated mineral particles (EMPs), originating from rocks and soils and once disturbed by natural erosion or anthropogenic activities, can be released into the atmosphere (Brook et al., 2020; Harper et al., 2017). While not all EMPs are hazardous, some are known carcinogens, such as erionite and some forms of naturally occurring asbestos (NOA). Less is currently known about the toxicity of other zeolitic EMPs, such as offretite and mordenite.

In environmental settings, different types of EMPs are often found closely located in the rock strata. Identifying which particle is present in the atmosphere is important to accurately quantify occupational and environmental risk to exposed populations. For example, erionite is classified as a Group I carcinogen (Vainio et al., 1991) and has been linked epidemiologically to malignant mesothelioma, with toxicological studies indicating that it is more carcinogenic than asbestos (Coffin et al., 1992; Dogan et al., 2008; Kraynie et al., 2016; Pacella et al., 2018).

Erionite was first identified in New Zealand in 1978 (Sameshima, 1978). Recent studies have confirmed the presence of erionite fibrils in rock close to the ground surface within Miocene Waitemata Group sediments, across several of Auckland’s urban locations (see Fig. 3) (Patel et al., 2022, 2024). Erionite has also been recently identified in rural areas of New Zealand’s South Island (Patel et al., 2024). However, other zeolites, which are morphologically and chemically very similar to erionite but are not known carcinogens, are often found co-located with erionite within New Zealand’s geological settings. Therefore, it is important to be able to accurately distinguish between EMPs detected in airborne samples, especially if erionite is present in the rock strata.

Several studies have been conducted in the USA, Turkey and New Zealand aimed at quantifying erionite in rock, soil and in air matrices (Beaucham et al., 2018; Berry et al., 2019; Carbone et al., 2011; Dogan, 2003; Farcas et al., 2017; Giacobbe et al., 2023; Talbot et al., 2022, 2024). Though erionite has been found in these environments and its carcinogenicity has been confirmed, erionite exposure levels (in ambient air) are not well understood nor have they been regulated (Fan et al., 2024). This is primarily because its presence is limited to highly localized areas of volcano-clastic geology, and current methods for detecting it in ambient air are tedious and time-consuming. Therefore, there are limited real-world investigations that explore the dose-response relationship of erionite in ambient settings (Dogan, 2003). Quantifying ambient concentrations of microfibers requires sampling a known volume of air onto filter paper, followed by a detailed analysis of the particles collected using advanced microscopic techniques. As reviewed by Shao et al. (2022), there are several advanced microscopic techniques such as optical, electron, and atomic force microscopy currently used to analyze the individual particle morphology of aerosols in air quality studies. Similarly spectroscopic techniques such as Raman Spectroscopy, Fourier Transform Infrared Spectroscopy, and X-ray diffraction (XRD) are used in chemical composition analysis and chromatographic and mass spectrometric techniques are used in identifying polymeric composition of particulate pollution in real-time. However, due to low concentrations of microfibers in ambient air samples, these techniques are unsuitable for detecting microfibers as particle numbers will likely be below the detection limits of most techniques listed above. Several approaches for testing air samples to quantify erionite concentrations have been documented in the literature (Beaucham et al., 2018; Berry et al., 2019; Carbone et al., 2011; Dogan, 2003; Farcas et al., 2017; Giacobbe et al., 2023; Talbot et al., 2022, 2024). These studies generally adapt existing asbestos quantification and characterization methods, such as the NIOSH 7400, NIOSH 7402 or ISO 10312 methods for asbestos. Beaucham et al. (2018) used Transmission Electron Microscopy with energy dispersive spectroscopy (TEM-EDS), following NIOSH 7402 and detected Erionite concentrations ranging from 0 to 0.36 fibers per cubic centimeter (f/cc) in the breathing zone of employees working in the Arikaree and White River rock formations in South Dekota and in the Custer-Gallatin National Forest in Montana. Carbone et al. (2011) reported fiber counts using TEM based techniques (peak concentration of 2.74 f/cc) and PCM equivalent values (PCME) (peak concentration 0.2 f/cc).

Studies that used PCM to count fibers greater than 5 μm in length and with a minimum length-to-width ratio of 3:1 following NIOSH 7200 for asbestos and the results are reported as structures per cm^3^. Although PCM is not able to identify fibers by specific mineral type, it serves as a quick fiber indexing approach for detecting elevated fiber counts, and therefore its presence or absence as a hazard. Samples reporting high fiber counts may subsequently be subjected to TEM-EDS analysis following the NIOSH 7402 standard for asbestos (Berry et al., 2019; Carbone et al., 2011; Farcas et al., 2017; Ray, 2020).

Erionite has a distinctive *d*-spacing (*c* =15 Å) compared to physically and chemically similar zeolite fibers (e.g. mordenite and offeretite), making it an ideal candidate for identification by TEM (Fan et al., 2024). However, erionite fibers are extremely sensitive to the high energy (~100 kV) electron beam used in TEM which can diffuse sodium and potassium and rapidly degrade the crystal lattice, complicating EDS elemental identification (Berry et al., 2019; Fan et al., 2024). Further, TEM analysis is expensive and time-consuming, requiring individual particles to be scanned manually. In environmental settings, where fibers may be present as a complex mixture within different samples, the small sample size required for detailed analysis, and the assumption that this represents the composition of all fibers counted using PCM, could result in both significant over or under-estimation of the fiber count and toxic component within the fiber composition (Snyder et al., 1987). In geological samples, techniques such as X-ray Powder Diffraction (XRPD) and Electron Probe Microanalysis (EPMA) are widely employed to determine the crystallographic structures and chemical composition of zeolites in geological studies, enabling the bulk samples to be identified. However, such techniques do not always lead to the determination of morphology and are less effective in the case of air sample analyzes due to the small amount of sampled material involved (Dogan, 2012).

Another approach to analyzing EMPs is to use a scanning electron microscope (SEM) to produce images of the fibers collected on filter material, followed by EDS using a low energy beam in the range of 10–15 kV, minimizing the damage to the lattice structure of the fibers (Carbone et al., 2011; Spurny et al., 1979; Talbot et al., 2022, 2024). SEM imaging and EDS analysis are promising approaches for determining the numerical concentration of EMPs. Existing ISO 14966-2019 and VDI 3492 provide detailed guidelines for air sampling, SEM scanning, and EDS analysis for asbestos fibers at detection limits of around 300 fibers/m^3^. To achieve good analytical sensitivity, the guidelines recommend manually analyzing an area of 1 mm^2^ on a 25 mm particle-loaded polycarbonate (PC) filter across a total filter area of approximately 385 mm^2^ (ISO - International Organization for Standardization, 2019)

The initial screening of filters using manual methods such as traditional PCM techniques or manual SEM imaging and EDS requires analyzing 50 to 400 fields of view (FOVs) for elongated mineral fibers. This is extremely time-consuming and requires significant labor and instrument time to complete. Given the relatively low level of fiber counts expected on air filters, there is a risk that the analysis of only a few FOVs of air filters could lead to false negatives due to poor analytical sensitivity.

Recent studies have been conducted in New Zealand where precautionary environmental sampling has been undertaken to determine the presence/absence of erionite fibers in air. These studies have highlighted the need for an automated process to analyze air samples collected on both filter papers and plant leaves to produce SEM images paired with EDS data, thereby enhancing throughput and analytical sensitivity in the detection, identification and quantification of erionite fibers (Fan et al., 2024; Talbot et al., 2024). Cho et al. (2011) has implemented an automated approach for asbestos fiber counting with PCM utilizing a motorized stage to capture multiple light images and coupling these images with image processing software for automatic fiber counting. Several studies have employed SEM-based automated methods to count particles or fibers on different substrates (Kim et al., 2020; More et al., 2011; Schulz et al., 2020; Stanger et al., 2014; Wilkinson et al., 2013), and have used automatically-generated EDS data for the identification of elemental composition of particulate matter within air samples (Neukirchen et al., 2024; Tiwari et al., 2024; Wagner et al., 2019; Wilkinson et al., 2011, 2013).

The novel methodology that is presented in this paper employs an SEM, equipped with a motorized stage for comprehensive particle analysis over a larger area of air filters in search of EMPs. This approach integrates SEM images with advanced particle analysis software that yields data on particle size and morphology and combines these with the automated collection of elemental composition through EDS analysis. The entire analytical workflow is programmed to identify particles based on their elemental composition and dimensions with a specific focus on detecting EMPs and also specifically erionite. The proposed methodology can assist risk assessment by establishing a framework for the automated, high-throughput analysis of air filters, potentially facilitating a more precise assessment of associated risks.

## 2. Materials and methods

The methodology for the automated counting of elongated mineral fibers and the classification of erionite fibers was developed using a JEOL JCM-7000 Neoscope^™^ (JOEL Limited, 2024) benchtop SEM with an automated stage (from here referred to as the JCM-7000). This SEM is equipped with an EDS detector and integrated with JEOL Particle Analysis Software 3 (from here referred to as PA3). To coat the segments of filters for SEM imaging and EDS analysis, a lab-grade gold sputter-coater (DII – 29030SCTR Smart Coater) was used. To test this method, a set of performance evaluation (PE) standard samples of known fiber concentrations were developed by spiking a known mass percentage of erionite onto PC filters using the methods described below. The erionite was obtained from Timber Bay, New Zealand, and its presence confirmed through SEM, XRD, and EMPA. The XRD results indicated that the sample contained 20 % erionite (EMPA formula ${Na}_{4.62}{\;K}_{1.70}{Ca}_{1.27}{Mg}_{0.51}{Ba}_{0.01}{\left[{Si}_{26.78}{Al}_{9.21}\right]}_{35.99}O_{72}29.07H_{2}O$ (Scarfì et al., 2024). The tested and validated method was then applied to real-world air samples collected on PC filters.

### 2.1. Preparation of PE standards

Previous studies have employed two distinct techniques for spiking mineral fibers onto air filters: the wet method and the dry method (Berry et al., 2019; Farcas et al., 2017; Kominsky et al., 2010). In the wet method, the fiber sample, along with any impurities, is washed off into deionized water and subjected to sonication. The supernatant is then filtered onto an air sampling filter and dried. The limitation of this approach is that the fibers tend to get stuck together in globules and are not dispersed on the filter paper in the same manner as would expect for air samples (See Fig. S1 of the Supporting Information). In contrast, the dry method involves fluidizing the sample in a fluidized bed, with air samples with fibers collected from the top of the bed (Berry et al., 2019). This method uses very specific apparatus unavailable outside of the USA.

**Fig. 1.**
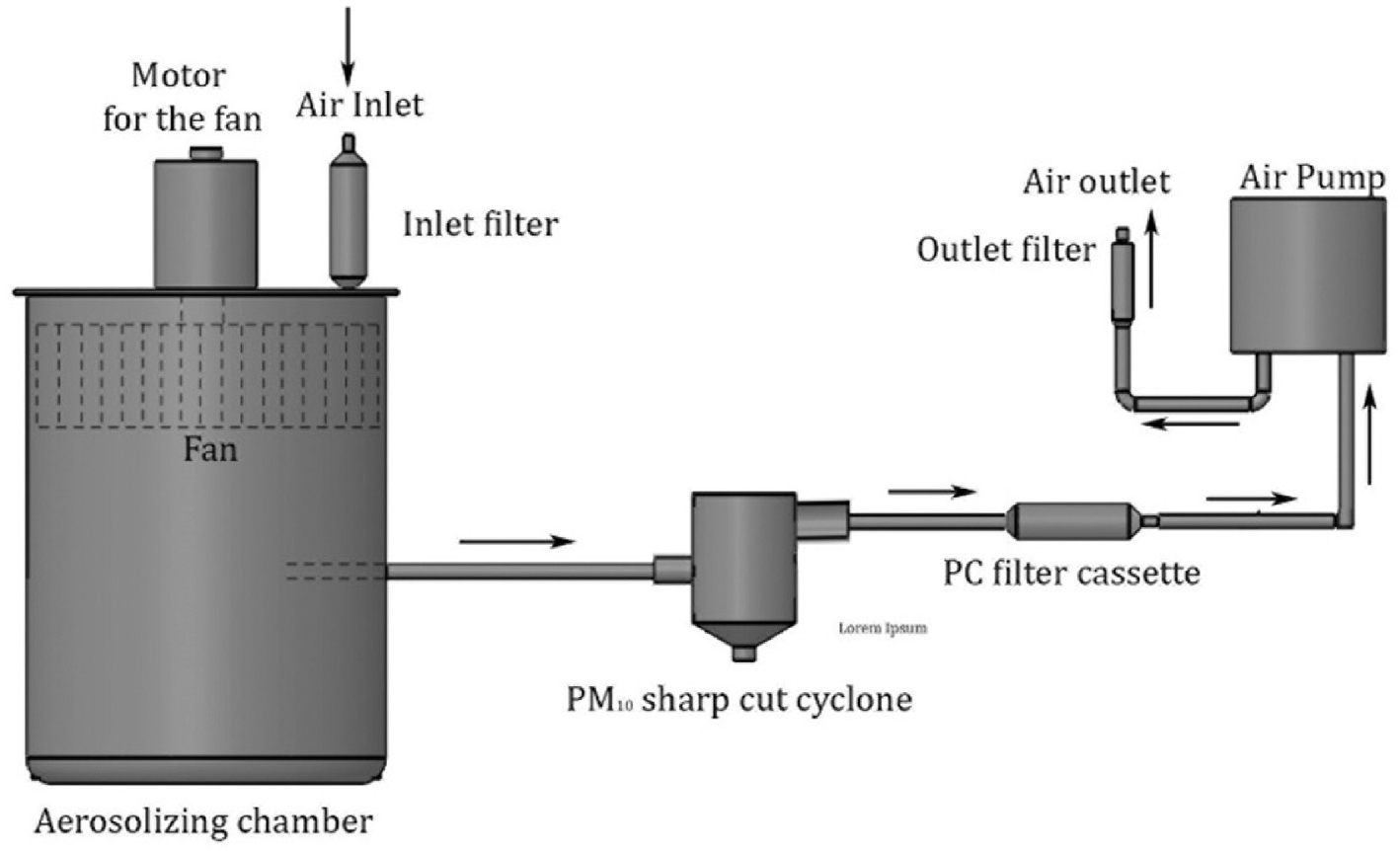
The apparatus used for particle aerosolization and air sampling.

The current study employs a novel dry spiking technique wherein a mixture of silica and erionite of known mass fraction is aerosolized within an aerosolizing chamber (designed and manufactured by Mote Ltd., Auckland, New Zealand) as shown in Fig. 1. The particles are aerosolized by mechanical agitation inside a closed chamber for 2 min. The air within the chamber is then sampled onto PC filters for 1 min at a flow rate of 2 LPM for successive automated SEM particle analyzes. A PM_10_ sharp-cut cyclone is installed between the aerosolizing chamber and the air filter cassette (See Fig. 1) so that particles larger than 10 μm do not get deposited on the air filter, as the intent was to sample the respirable fraction of fibers. The aerosolizing and sampling times required to achieve an acceptable particle distribution were determined through trial runs. The flow of air that passes through the PC filter is then sent through a second filter to avoid erionite fibers being released into the atmosphere with the apparatus operated under a fume hood. To prevent cross-contamination, the aerosolizing apparatus was thoroughly cleaned between runs.

A series of bulk samples were prepared by mixing 3 g of silica with a known mass of erionite sample obtained from Timber Bay, New Zealand described above. The silica used here is microcrystalline quartz from silicified Miocene lacustrine sediments from the Coromandel Peninsula, New Zealand. Since samples from Timber Bay have previously been determined to contain 20 % erionite by mass, the procedure allowed for the production of a series of bulk samples with erionite concentrations of 0 %, 0.02 %, 0.15 %, 0.37 %, 0.89 %, and 1.7 % by mass, respectively (named PC1 to PC6). These samples were then aerosolized using the apparatus described in Fig. 1 and collected onto pre-weighed PC filters with a pore size of 0.8 μm. After aerosolization and sampling, the filters were re-weighed before being prepared for SEM imaging, particle counting and EDS analysis.

Additionally, one sample containing 20 % erionite fibers (named SPC1) was prepared by spiking 0.02 g of the Timber Bay erionite sample directly, without mixing with silica. This filter was used to determine the optimal SEM magnification for fiber counting and EDS analysis during performance evaluation.

### 2.2. Programming automated fiber counting

The first step in automated particle counting and analysis using the JCM-7000 and PA3 particle analysis software involves configuring the method. This process consists of four key steps: setting the particle identification conditions, selecting the analysis region, configuring elemental analysis settings, and defining the particle classification criteria.

To establish the particle identification conditions, the sample is focused with the desired magnification, and the brightness and contrast are adjusted to distinguish particles from the PC filter substrate. Membrane filters, such as mixed cellulose ester (MCE) filters, are not recommended for this purpose due to their spongy surface structure which complicates the counting process. Additionally, smaller fibers tend to penetrate deeper into these filters, making them harder to detect. In contrast, polycarbonate nucleopore filters have a flat, clean surface making them well-adapted for this purpose (See Fig. S2 of Supporting Information).

Once the region of interest has been set and binarization has been performed, the brightness and contrast settings are then fixed, saved, and maintained consistently across all FOVs during analyzes. Since the aim is to count and classify respirable elongated mineral fibers, fibers with an aspect ratio of at least three and a width of less than 3 μm are selected for counting, consistent with asbestos counting standards NIOSH 7400 (Cassinelli and O’Connor, 1994). However, the length criterion of greater than 5 μm was not adopted here as Fan et al. (2024) previously identified erionite fibers shorter than 5 μm in length in air samples collected in New Zealand. After detection, the FOVs to be analyzed are chosen and saved.

When obtaining an EDS spectrum for individual particles using the manual approach, the full EDS spectrum is acquired and presented (Berry et al., 2019; Fan et al., 2024; Farcas et al., 2017; Patel et al., 2022; Talbot et al., 2022, 2024). However, the carbon and oxygen peaks in the EDS spectrum are influenced by the carbon and oxygen present in the PC filter, leading to mass percentages of carbon and oxygen that do not accurately reflect the true composition of the analyzed particles. Therefore, only the mass percentages of other elements commonly found in zeolites, such as Si, Al, Mg, Na, K, Ca, Fe, and Cl, are considered, as this also helps to reduce analysis time significantly. These percentages are used to calculate the Tetrahedral Tsi ratio (Si/(Si + Al)) to allow for the differentiation of erionite from other zeolitic species. The mass percentages of the extra-framework cations were also used to compare erionite fibers with other zeolite particles (in line with the analysis presented by Carbone et al., 2011). As reported by other researchers, SEM-EDS analysis provides qualitative or semi-quantitative information regarding elemental composition, with the Tsi ratio alone being insufficient to allow the crystallographic structure of different fibrous zeolites to be distinguished (Fan et al., 2024). Nonetheless, in this study, it served as an initial screening technique for differentiating erionite-like structures from other zeolites, with further analysis required to confirm and distinguish erionite from other types of EMPs.

The next step in the automated analysis involved setting the EDS analysis time. For individual particle analysis, an analysis time of 30–60 s is typically used (König, 2023). However, to increase the throughput, an analysis time of 10 s was adopted here. To verify the accuracy of the EDS spectrum obtained using an analysis time of 10 s, experiments were conducted on standard copper, an erionite fiber, and a salt particle, with analysis times of 10 s, 30 s, 60 s, and 100 s, respectively. For all these samples, including erionite, the results showed that although the intensity counts decrease with shorter analysis times, all relevant peaks can still be identified using a 10s analysis time. This demonstrates that no elements are missed in the spectrum and that there is no effect on the Tsi ratio (see Figs. S6, S7, and S8).

**Fig. 2.**
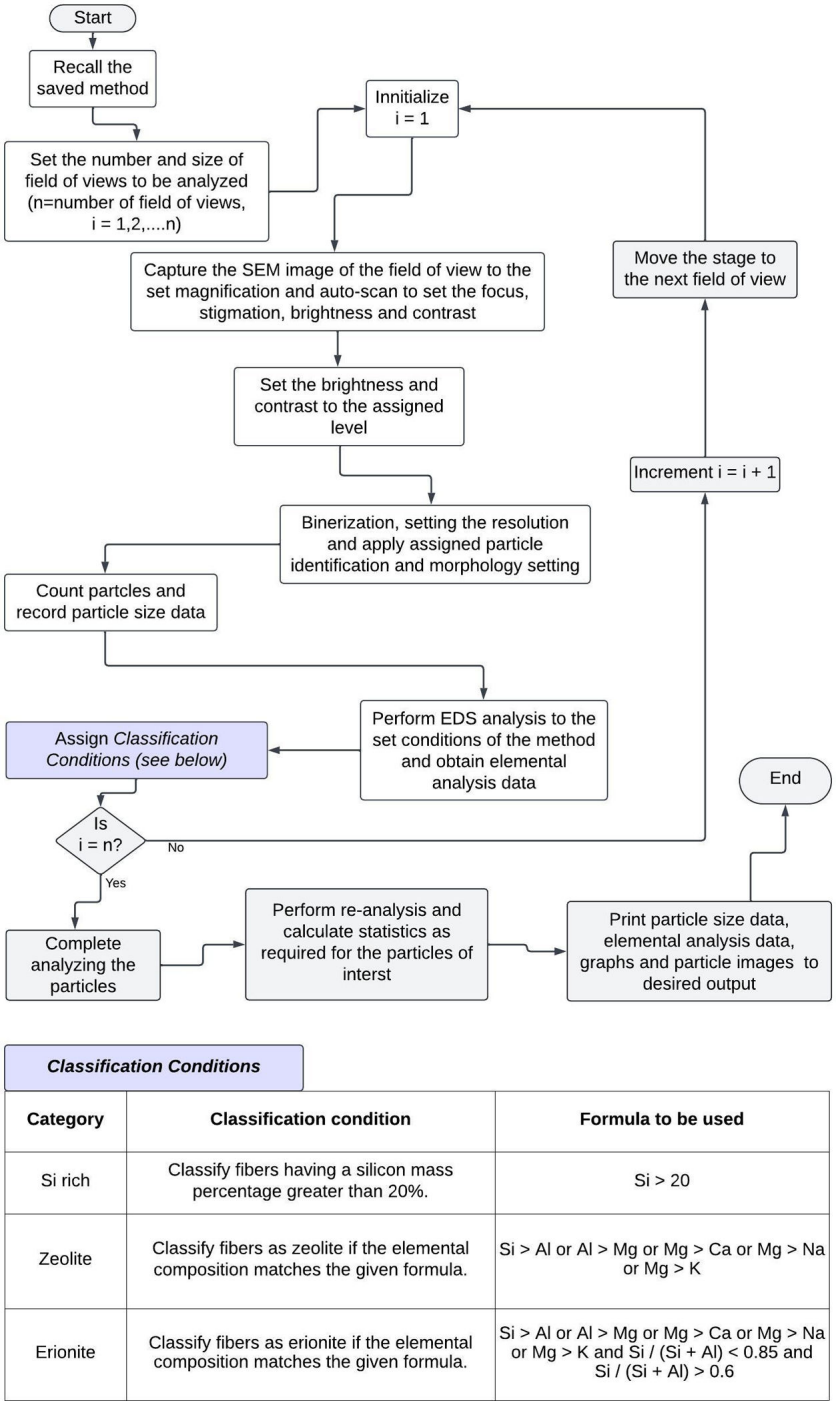
Workflow of the automated particle analysis.

The particle *classification criteria* are outlined within the workflow of Fig. 2 and then saved for automated counting, after which it may be recalled and applied as needed. Upon completion of the counting and classification, all data are saved in a spreadsheet.

A segment of the SPC1 filter (spiked with 20 % erionite without silica mixing) was mounted on an SEM stub using carbon tape and sputter-coated with a 20 nm layer of gold to determine the optimal SEM magnification. Automated fiber counting was conducted on a backscattered SEM image at an acceleration voltage of 15 kV at magnifications of 400X, 1000X, and 2000X, with a working distance of 13.4 mm. These magnification settings were selected to optimize the balance between maximum fiber count, analysis time, and accuracy.

PCM, commonly used in asbestos analysis, employs a standard magnification of 400X (Cassinelli and O’Connor, 1994). However, at higher SEM magnifications, the FOV becomes smaller, enabling the detection of even smaller fibers. Despite this advantage, higher magnification significantly increases the time for analysis using the automated process. To compensate, the area of the filter analyzed must be reduced, which in turn lowers the analytical sensitivity. A magnification of 2000X was used as the upper limit, as it provides sufficiently detailed images for particle analysis and EDS analysis while ensuring that analysis is able to be completed in a manageable amount of time. Moreover, this magnification is recommended by ISO 14966-2019 as this is the level of magnification required for fibers above 0.2 mm to be able to be viewed. A 1000X magnification was chosen to evaluate the SEM’s performance between these two extremes.

For each magnification setting, three trials were conducted using the same field of view to assess the repeatability of the fiber counts. The optimal magnification was selected based on both the fiber counts obtained and the time required for automated counting and analysis.

### 2.3. Comparison of fiber counts using ImageJ

The total fiber counts obtained on an air-sampled filter using the integrated particle analysis software of the desktop SEM were verified by performing fiber counting using ImageJ, a Java-based open-source image processing program. Several other researchers have successfully used ImageJ software for fiber counting with reliable results reported (Cho et al., 2011; Cossio et al., 2018). ImageJ particle analysis follows a similar procedure to automated SEM counting, where images are transferred into binary images based on certain algorithms.

### 2.4. Fiber counting, EDS analysis, and estimation of fiber concentration

A segment of each 25 mm circular polycarbonate filter from the PE standards was mounted onto an SEM stub using carbon tape and sputter-coated with a 20 nm layer of gold. The remaining portions of the filters were archived for future analysis.

Automated fiber counting, EDS analysis, and fiber classification were performed at 2000X magnification with an accelerating voltage of 15 kV and a working distance of approximately 13 mm. FOV were selected along a transect starting from the center of the filter to minimize the effect of an uneven particle distribution on the true mean count. Termination criteria, as described in ISO 14966-2019 (Refer to Extract-X in Supporting Information), were applied to determine the number of FOVs to be analyzed, maintaining the error limit at a minimum. Depending on the fiber concentrations observed on the PC filters, the number of FOVs analyzed was 25 for the spiked samples and 100 for the real-world samples.

### 2.5. Mean fiber count and limit of detection in fibers per cm^3^

The mean fiber count in fibers per cubic cm^3^ (f/cc) is estimated based on the analytical sensitivity adopted. The analytical sensitivity depends on the sampling and analysis parameters such as the airflow rate, sampling duration, total filter area and the area of the filter analyzed through the SEM. The analytical sensitivity can be described mathematically as shown below (ISO - International Organization for Standardization, 2019).

$$\mathrm{Analytical}\;\mathrm{sensitivity}=\frac{A_{\mathrm{effect}}}{n\cdot A_{FOV}\cdot v\cdot t}$$

where.

$A_{\mathrm{effect}}=$ Effective total filter area in mm^2^

$A_{FOV}=$ Area of a field of view being analyzed in mm^2^

n= number of FOVs being analyzed

v= Air flow rate in cubic centimeters per minute

t= time of sampling in minutes

Analytical sensitivity can be interpreted as the concentration in f/cc corresponding to a count of one fiber in a sample. Hence a fiber count obtained by analyzing n FOVs can be converted to a concentration in f/cc by multiplying the fiber count, N, by the analytical sensitivity.


Fibreconcentration(f/cc)=N.AnalyticalSensitivity

The accuracy of the estimated fiber concentration relative to the true mean concentration is highly dependent on the analytical sensitivity. A greater analytical sensitivity yields an estimated concentration closer to the true mean. The analytical sensitivity can be optimized by increasing the sampled air volume and expanding the analyzed filter area. While increasing the sampled air volume generally enhances fiber detection by raising the fiber count, it can also lead to filter overload with non-fibrous materials such as dust and organic matter, complicating fiber identification. Unlike manual SEM imaging and EDS analysis, as outlined in ISO 14966-2019 (ISO - International Organization for Standardization, 2019), the automated approach proposed in this study enables control over the filter area analyzed. However, determining the optimal filter area analyzed and the analytical sensitivity must consider the time allocated for analyzing a single air filter. Depending on the number of filters processed from a given air sampling activity, a trade-off between the analytical sensitivity and analysis time per filter may be necessary. The advantage of the automated process is that these adjustments can be made so that the analysis can be performed without human intervention, thereby maintaining both efficiency and consistency.

The limit of detection (LOD) is defined as the fiber concentration below which the true concentration falls with 95 % confidence when no fibers are observed during scanning electron microscope (SEM) examination. The LOD for the proposed method was evaluated using the prepared PE standards.

When the numerical fiber concentration on the air filters is very low, the probability of detecting fibers on a given air sampled filter by analyzing a number of FOVs can be modeled according to a Poisson distribution, as described in ISO 14966-2019 and ASTM D6620. On the basis of the Poisson distribution, using Tbl 4 of ISO 14966-2019, the 95 % confidence interval for the estimated fiber counts is calculated.

### 2.6. Real-world air sample collection and analysis

**Fig. 3.**
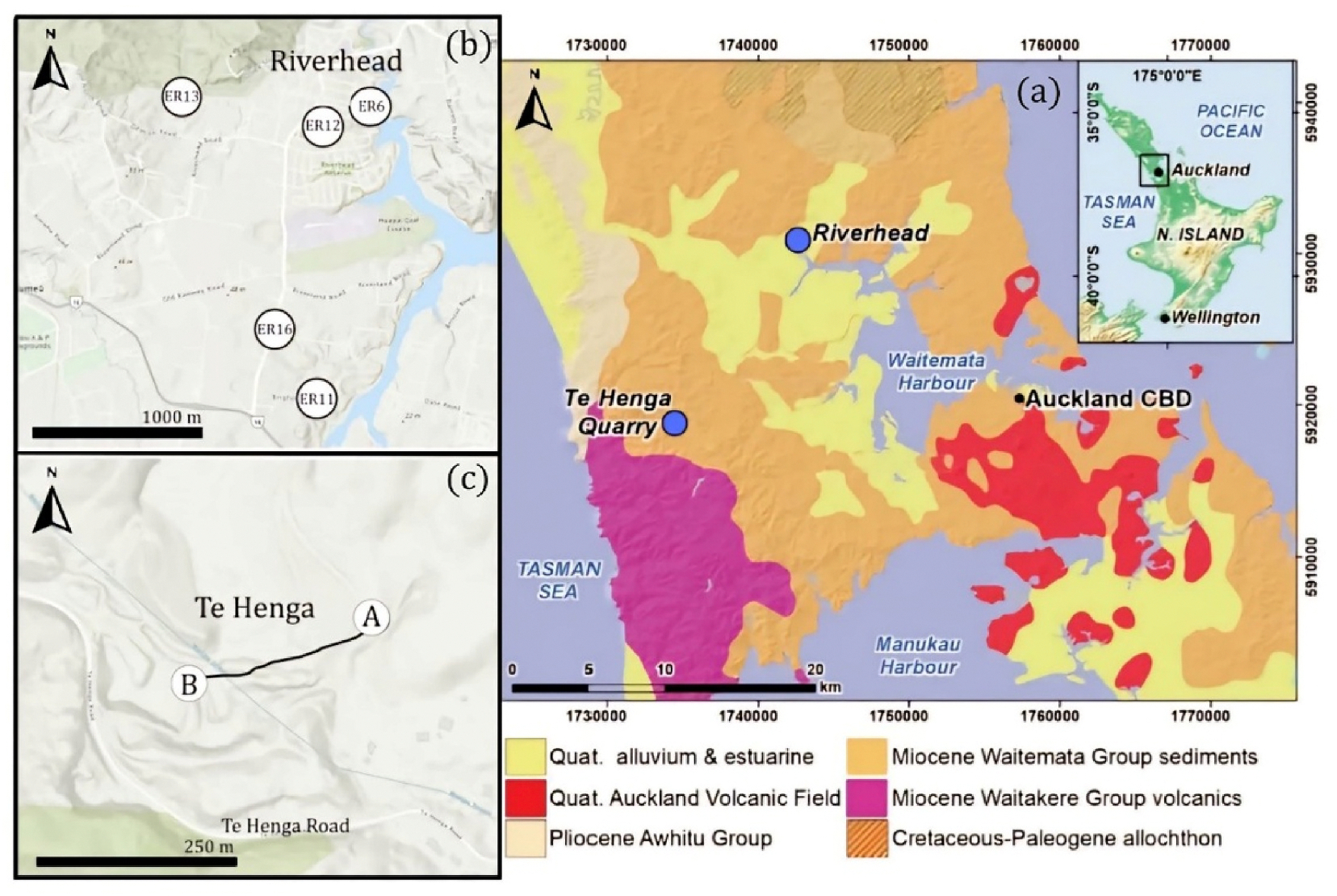
a) The map of Auckland region illustrating areas where Erionite-bearing rocks are present along with the two sampling sites Riverhead and Te Henga marked in blue, (b) Riverhead static sampling sites, (c) Te Henga mobile sampling route. (For interpretation of the references to colour in this figure legend, the reader is referred to the Web version of this article.)

Real-world air sample filters were collected across Auckland, New Zealand, where erionite-bearing rocks are present, from two sampling campaigns: one fixed-site, community monitoring campaign, and one mobile monitoring campaign (Talbot et al., 2024). A simple, portable sampling device was used for this work (the FilterMote (Patel et al., 2024), see Fig. S3), with samples collected on a seven-day schedule for the fixed-site campaign. During the mobile monitoring campaign, samplers were attached to the outside of a vehicle whilst it traversed a pre-determined route for varying sampling periods. The instrument sampled air at approximately 3.0 L min^−1^ with flows recorded during sampling and both validated and calibrated pre and post-sampling with a calibrated flow meter (Alicat MWB Series Mass Flow Meter). Samples were collected onto 25 mm PC filters with field and travel blanks used throughout the campaigns. After sample collection, filters were stored in sealed cartridges, at room temperature, away from light. A map presenting the sampling locations is presented in Fig. 3. The same methodology (outlined in Section 2.5) was also applied to the real-world samples for automated fiber counting, EDS analysis, and the classification of fibers. Table S1 presents the sampling locations, sampling times, flow rates, and volumes used in the campaigns.

## 3. Results and discussion

### 3.1. Selection of suitable SEM magnification

**Fig. 4.**
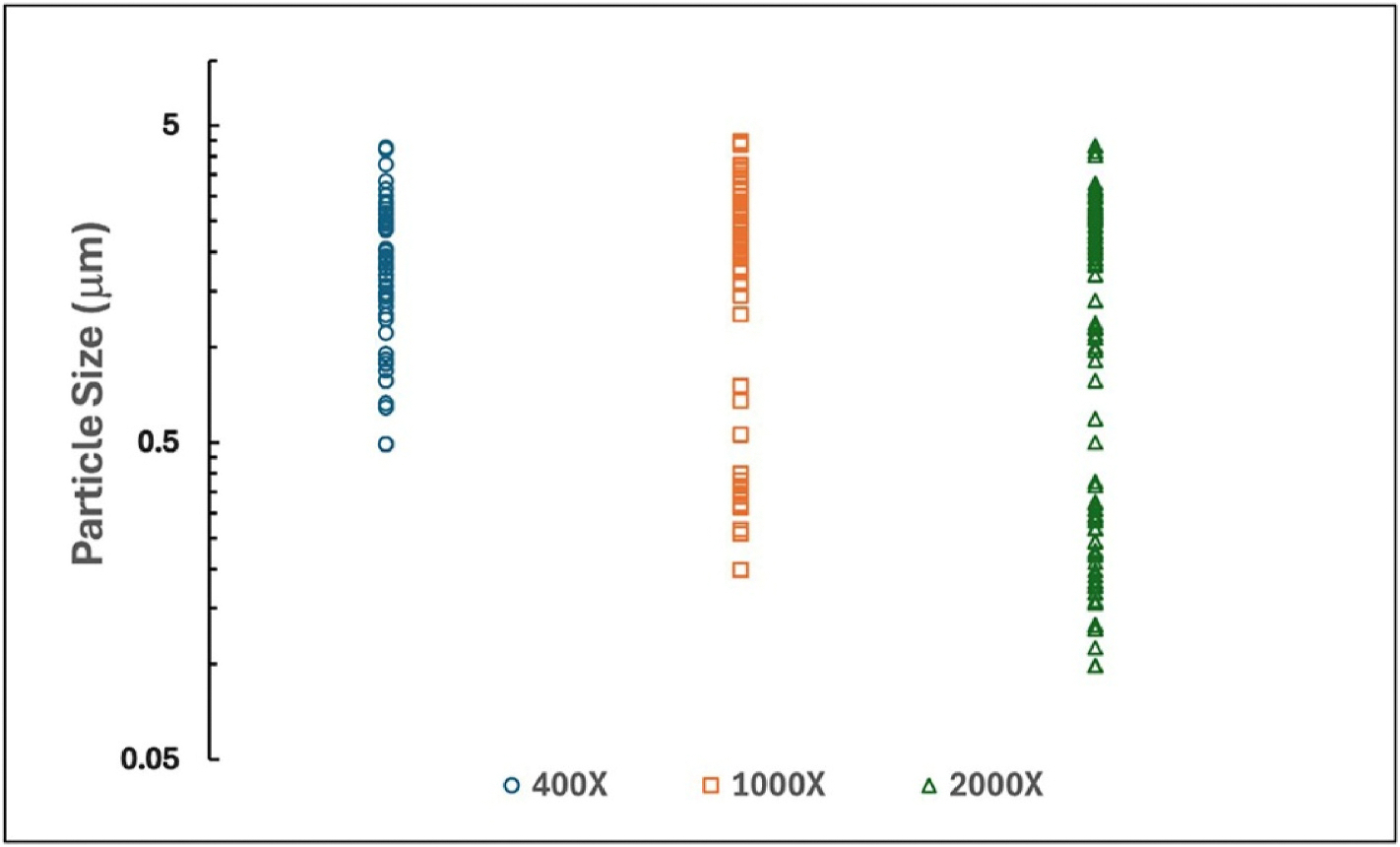
The particle size distribution of trials conducted on the same FOV at 400X, 1000X, and 2000X magnifications. Note that the y-axis is a log scale.

Fig. 4 illustrates the outcomes of three repeated trials conducted on the same size of FOV (filter area of 0.003 mm^2^) at magnifications of 400X, 1000X, and 2000X. Particle counting and classification were carried out to identify erionite fibers. It was observed that fiber lengths vary from 0 to 5 μm. The particle size distribution suggests that an increasing number of smaller particles are detected as the magnification is increased. Fiber counts recorded under magnifications of 400X, 1000X, and 2000X were 36(±4), 53(±11), and 128(±24), respectively. The analysis duration for each magnification for the same filter area increased from 15 min at 400X to over 3 h at 2000X. The observed higher fiber counts at increased magnifications are attributable to the enhanced resolution of the particle boundaries, allowing fiber bundles, often counted as single fibers at lower magnifications, to be resolved into individual fibers at higher magnifications. Furthermore, the reduced field of view at 2000X magnification ensures that even smaller fibers in the range of zero to 0.5 μm in length are detected, owing to the smaller pixel size, in line with ISO 14966-2019.

These results indicate that the proposed method is effective for fiber identification amongst fibers that meet the classification criteria. However, to ensure accurate fiber counts per cm^3^, it is recommended that analysis be conducted at high magnification, such as 2000X, which provides both a manageable analysis time and an increased level of fiber detection accuracy.

### 3.2. Comparison of fiber counts of SEM software (PA3) with ImageJ

Fig. S4 shows the correlation between the length parameters of 308 fibers in a single field of view of a road sample, as measured by SEM software (PA3) and ImageJ software. The results indicate a strong correlation between the counts obtained from the two methods. However, the ImageJ software tends to underestimate the length parameter slightly compared to PA3 (See Fig. S4 showing the regression line underestimates by 7.58 % compared to perfect correlation). This discrepancy could result from differences in how the two methods define particle boundaries. Nevertheless, a calibration curve can be used to correct the length differences estimated between the two methods.

### 3.3. Analysis of performance evaluation standards

**Fig. 5.**
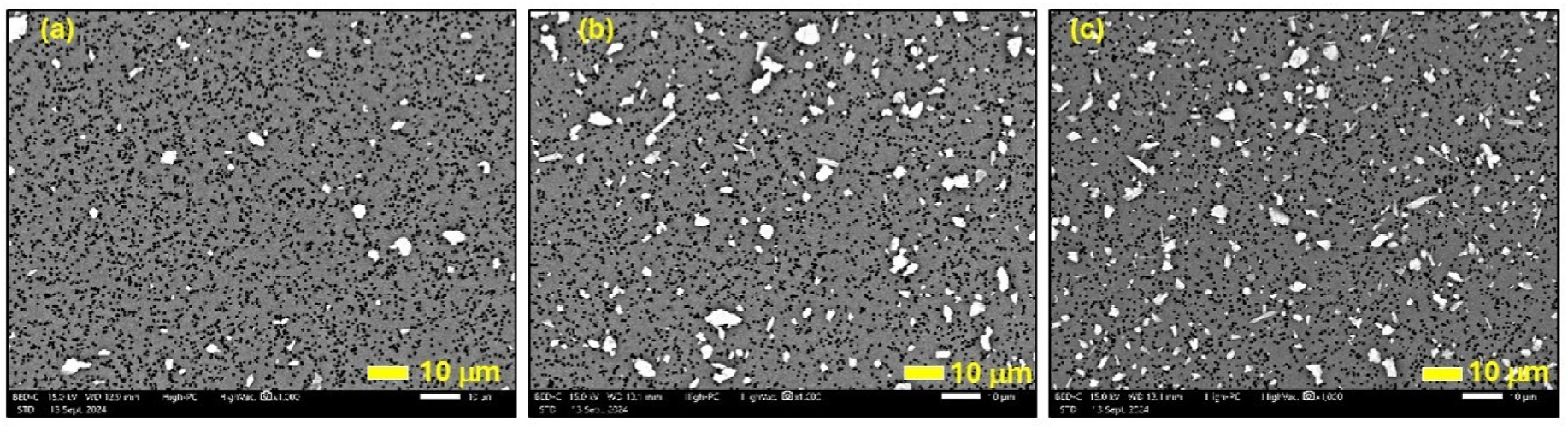
SEM images at 1000X magnification of (a) PC2 (0.02 % erionite), (b) PC3 (0.37 % erionite), and (c) PC6 (1.74 % erionite).

**Fig. 6.**
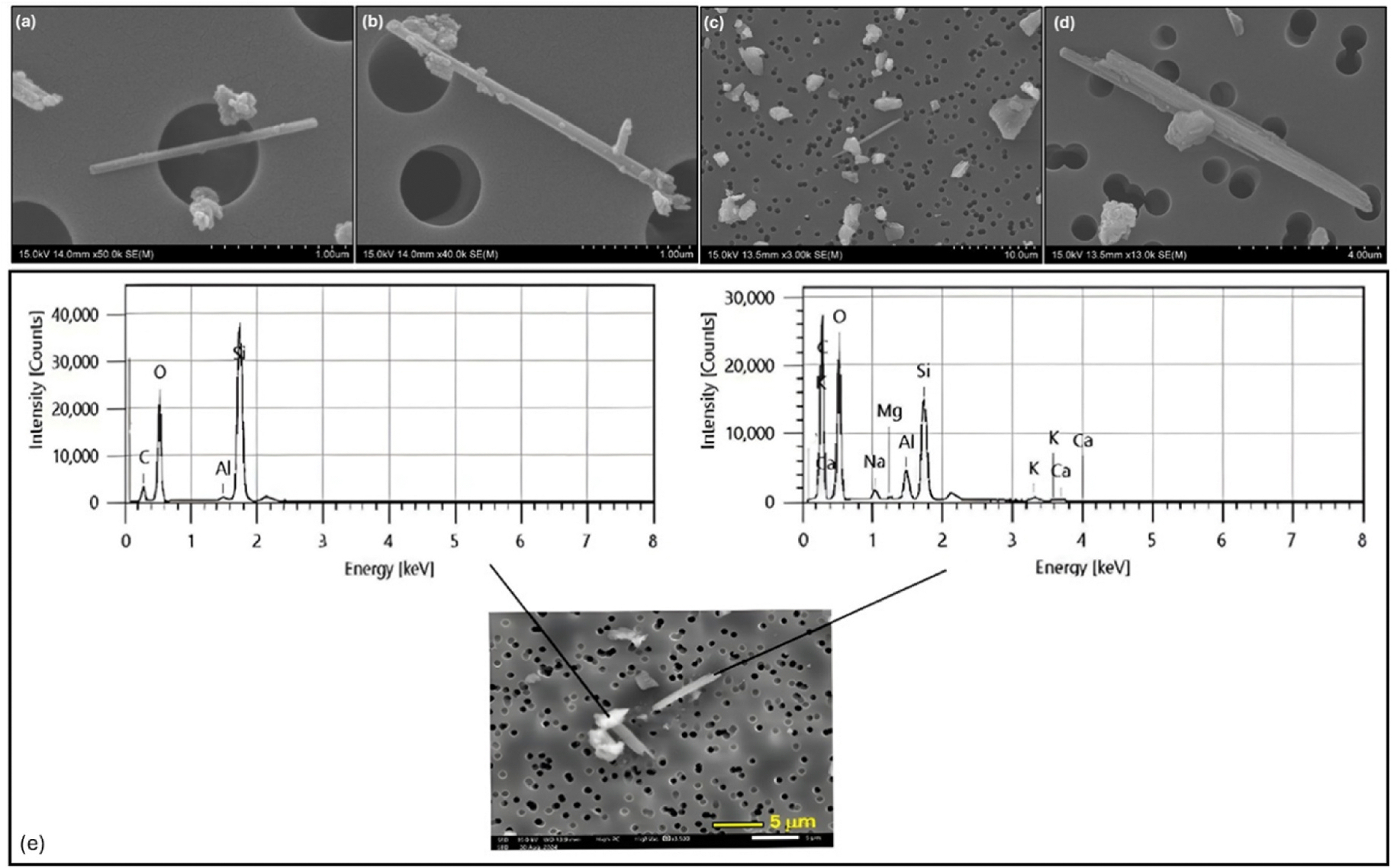
Higher magnification images illustrating erionite fibers on PE standard filters. (a) and (b) single fibers and (c), (d) fiber clusters and (e) EDS analysis of Silica and Erionite showing the difference in elemental composition. The unlabelled peak around 2.1 keV is a result of the gold sample coating. (For interpretation of the references to colour in this figure legend, the reader is referred to the Web version of this article.)

Fig. 5 presents SEM images at 1000X magnification of PC2 (0.02 % erionite), PC3 (0.37 % erionite), and PC6 (1.74 % erionite), respectively. The images demonstrate differences in the concentrations of elongated mineral fibers on the PC filter, suggesting that mechanical agitation occurring within the apparatus aerosolized fibers in proportion to their mass loading. However, it is also observed that the fiber lengths are below 5 μm and that the mechanical agitation has fragmented fibers into small sizes. Higher-magnification images were captured using a floor-standing SEM (Hitachi SU-70 Schottky field emission SEM), as shown in Fig. 6. It further illustrates the presence of individual fibers (Fig. 6(a) and (b)), while clusters of fibers are also visible (Fig. 6(c) and (d)).

In addition to the images above, EDS analysis of two different types of particles on the PE standards confirms the presence of a silica and erionite mixture on the PC filter, as shown in Fig. 6(e). According to the classification criteria applied during the automated process, erionite and other zeolites in the sample are classified based on the Si/(Si + Al) ratio (as per Fig. 2), allowing them to be distinguished from each other.

The results obtained by applying the methodology described above to all PE standard filters (PC1 to PC6) are presented in Table S2. The results from five different transects (each transect has five FOVs at 2000X magnification) of the same segment of the filter paper were analyzed to determine the total fiber count per total FOV analyzed at 2000X magnification. The 95 % confidence interval of the mean has been calculated based on ISO 14966-2019 assuming a Poisson distribution (recommended for the random sampling of FOVs from the whole of the filter, as given the ISO 14966-2019 standard). The higher range of confidence interval at high mass percentages of erionite, such as the observed mass percentage of 1.74 %, is due to increased sample loading when analyzing spiked samples which have higher fiber counts. When different transects are analyzed, the fiber counts vary (larger sample size gives larger variability), and this variation increases with the mass percentage of erionite in the bulk sample.

**Fig. 7.**
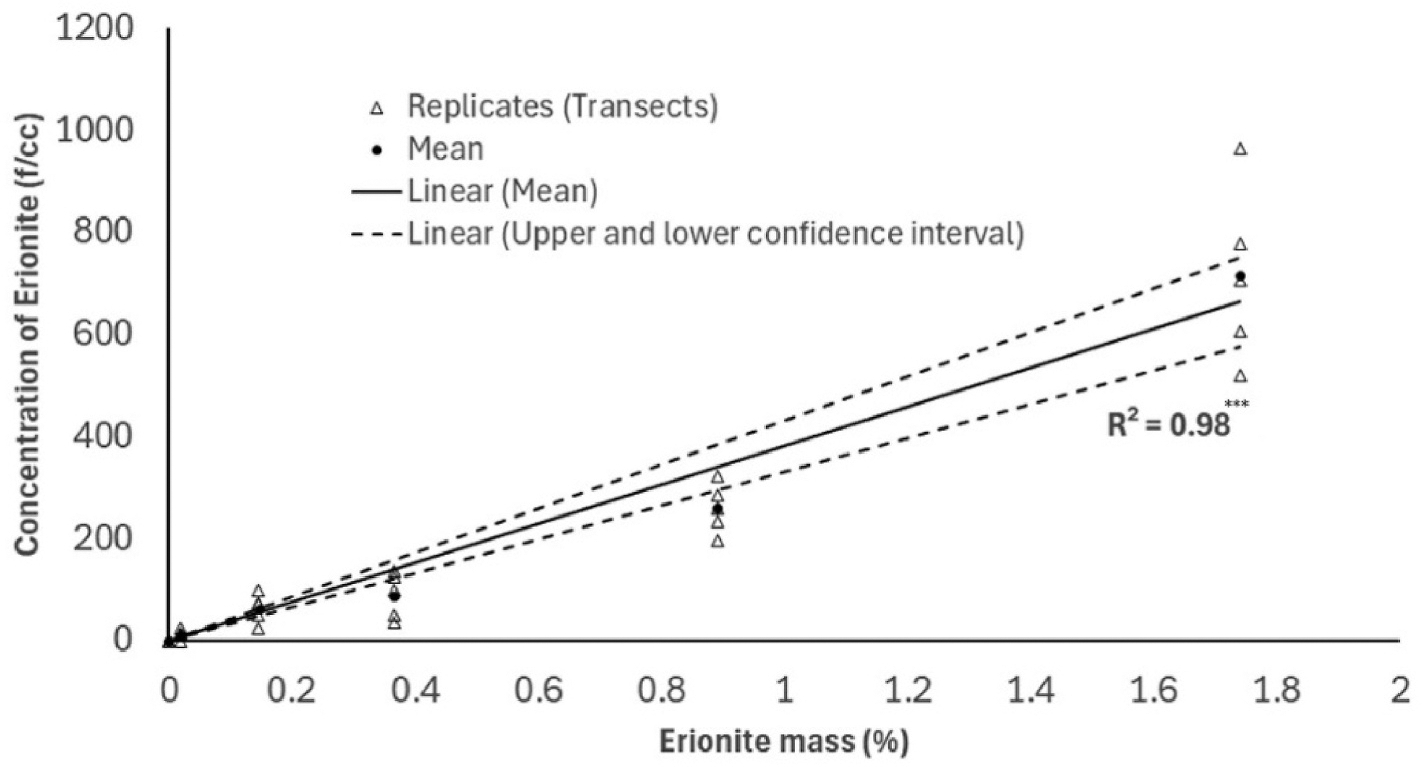
Results presenting estimated erionite fiber counts per cubic centimeter of aerosolized air volume against the erionite mass percentage in the bulk sample. *** Significance level p < 0.001.

These fiber counts were used to calculate the number of fibers per cm^3^ (f/cc) of aerosolized air inside the aerosolizing apparatus using the calculated analytical sensitivity, as described above. The fiber counts per cm^3^ of air are plotted against the mass percentage of erionite in each PE standard and are shown in Fig. 7. The correlation between the mass percentage of erionite and the estimated fiber counts correlates with an R^2^ of 0.98 (p < 0.001).

Based on the analytical sensitivity applied to the spiked samples, a detection limit of 7.4 f/cc can be achieved. However, increasing the analysis area or sampled air volume can significantly improve this detection limit. To assess this improvement, PE standards of erionite at much lower concentrations would need to be prepared. Previous studies that have employed PCM, SEM, and TEM techniques to determine erionite fiber concentrations in ambient air have reported concentrations as low as 0.01–5.6 f/cc (Carbone et al., 2011; Dogan, 2012; Wolfe et al., 2017). A high detection limit was obtained for our PE standards due to the low sensitivity resulting from the low volume of air sampled to create the PE standards.

### 3.4. Real-world sample analysis

**Fig. 8.**
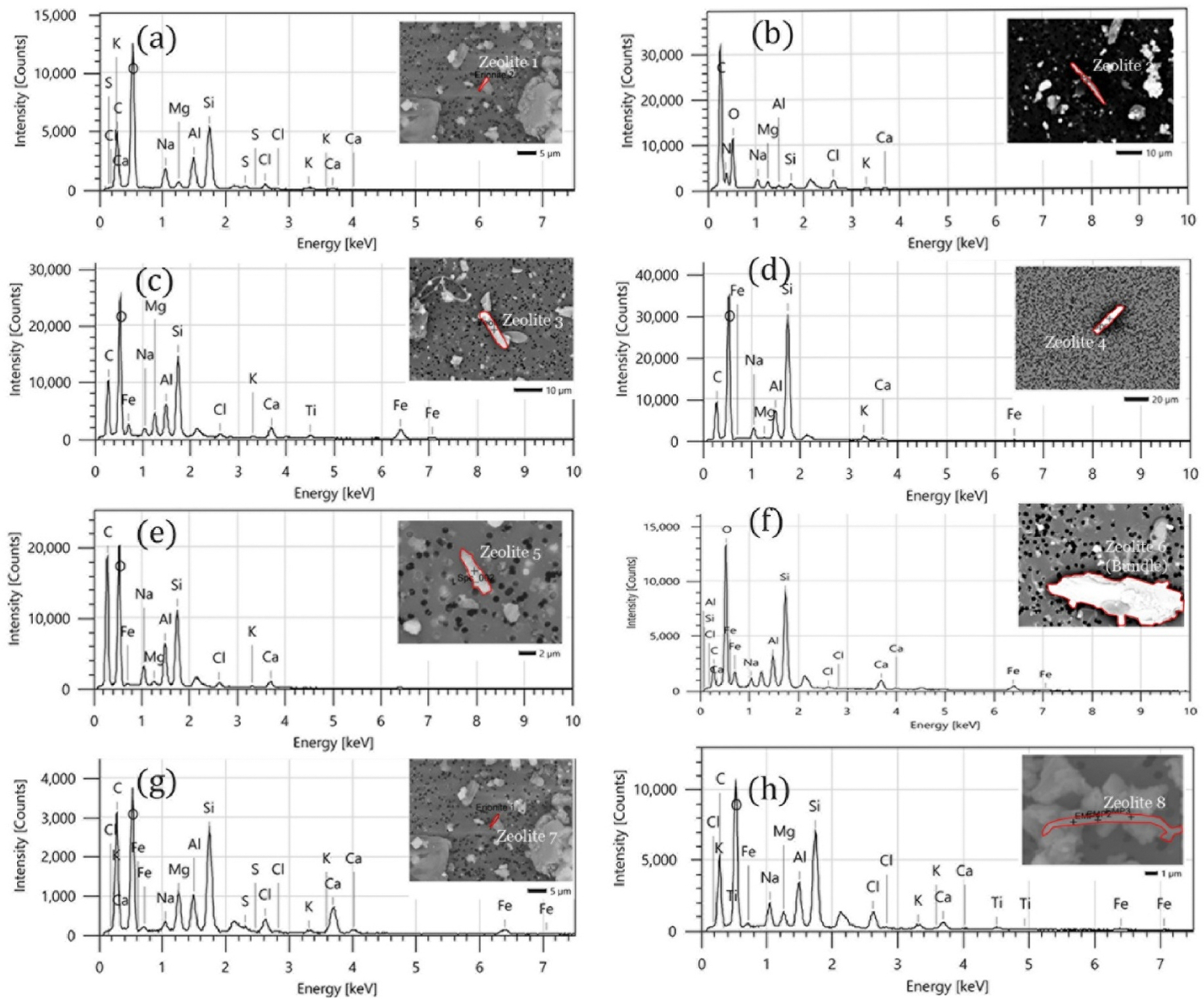
Elongated mineral fibers on real-world Riverhead and Te Henga samples (marked in red). Their site codes are as follows; [11(a) ER6], [11(b) ER11], [11(c) ER12], [11(d) ER13], [11(e) ER13], [11(f) ER16], [11(g) A-B]. The un-labelled peak around 2.1 keV is a result of the gold sample coating. (For interpretation of the references to colour in this figure legend, the reader is referred to the Web version of this article.)

SEM images and EDS data of various fibers, identified in real-world samples through an automated approach, are presented in Fig. 8. Although these fibers cannot be definitively confirmed as erionite, the findings are promising. Despite other particulate matter, such as road dust, marine aerosol, and biological material found on the air filters, the brightness and contrast adjustments of the SEM images enable the isolation of these particles and the acquisition of EDS data. Once elongated mineral fibers with an elemental composition similar to erionite have been identified using this screening method, a secondary technique such as TEM can be employed to confirm the presence of erionite by performing selected area electron diffraction (SAED). Erionite can be distinctly identified from other similar zeolite mineral fiber types based on distinctive d-spacing (c = 15 Å) of the crystalline structure for Erionite (Fan et al., 2024). Table S3 details the estimated time taken by the automated method to analyze different numbers of FOVs by scanning a backscattered image at 2000X magnification, an accelerating voltage of 15 kV and an EDS analysis time of 10 s for various filter loadings, providing a basis for decision-making based on specific requirements. The data presented in Table S3 were obtained by running the PA3 software of the Desktop SEM for several environmental samples. However, this time can vary depending on the number of particles of a specified size and aspect ratio available on the filter paper.

**Fig. 9.**
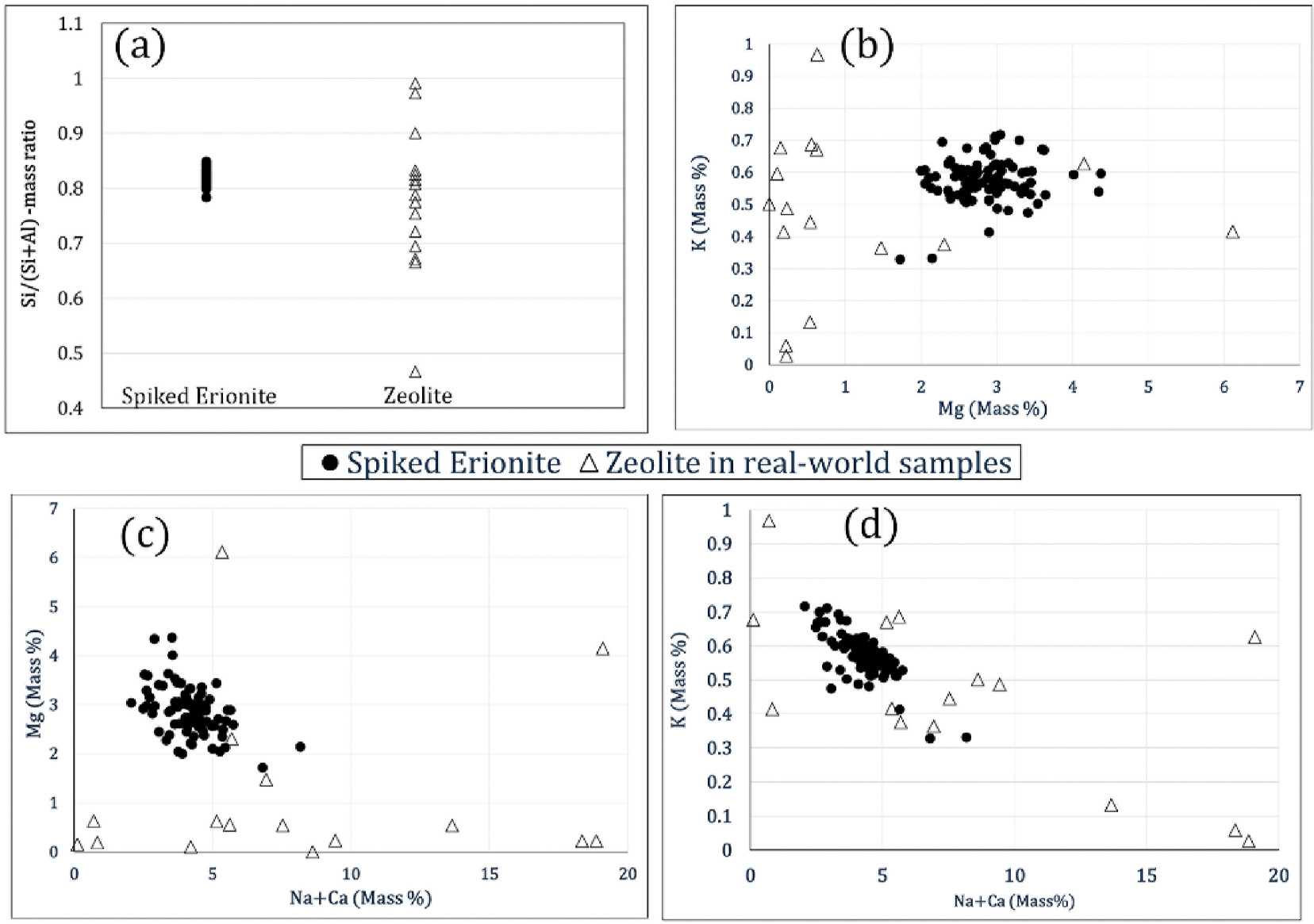
Comparison of elemental composition of erionite fibers identified through the automated process with zeolite fibers detected in air samples.

Elemental analysis results obtained through the automated process on the spiked filter at 2000x magnification were compared to those from real-world samples. The chemical data are presented in Fig. 9. As shown in Fig. 9(a), the average Tsi ratio of erionite fibers identified through the automated analysis aligns with expected values, confirming the findings of previous work (Carbone et al., 2011; Fan et al., 2024). This demonstrates the accurate identification of erionite fibers and the reliability of the elemental data from the automated process. Zeolite fibers identified in the road-sampled air filters did not consistently exhibit the Tsi ratio characteristic of erionite, except for very few fibers. However, according to Fan et al. (2024), the Tsi ratio of erionite and offretite are similar, requiring further analysis to distinguish between different mineral types. Additionally, the compositions of other elements, such as Mg, Na, K, and Ca, are compared and presented in Fig. 9(b), (c), and 9(d), in line with the analysis presented by Carbone et al. (2011). When the mass percentages of these elements were plotted, the data points of erionite are found to cluster together, indicating consistent elemental ratios and suggesting the potential for identifying erionite fibers against other zeolites during this initial screening process based on the elemental composition generated by the EDS analysis.

### 3.5. Limitations of the proposed method

Although the proposed screening technique shows promising results, it has some limitations. Air sampling needs to be carefully undertaken to avoid overloading the filter which could obscure any underlying fibers in the SEM image. If the filter is heavily loaded and if the particles are overlapping or cluster together, it is difficult to process the SEM image for fiber counting (see sample images in Fig. S5 of the supporting information). In this study, an air sampling flow rate of 3.3 L/min was used, with sampling times ranging from 30 min (mobile sampling) to one week (static sampling). The ideal sampling duration and the flow rate for obtaining high-quality SEM images for fiber counting depends on the specific objective and environmental setting of the research. The sampling time and flow rate should be adjusted based on the ambient particulate concentration, the filter media used, and the type of analysis.

During the automated process, it is difficult to ensure that the electron beam remains stable and properly focused on the fiber without drifting. If the beam drifts, some fibers may go undetected. Additionally, ensuring that the point of incidence of the electron beam is sufficiently far from adjacent or attached particles is challenging. If the beam intersects nearby particles, it can interfere with the spectrum, complicating the analysis. Having the filters moderately loaded can improve performance of the automated analysis. A thick sputter coating may also obscure some of the peaks in the EDS spectrum, leading to inaccurate fiber classification. Therefore, the sputter coating should be applied at a thickness sufficient to prevent charging during SEM imaging (a 20 nm thickness worked well in this study).

The method is designed to identify only straight fibers or fiber bundles that meet specific aspect ratio and elemental composition criteria, potentially missing fibers attached to other particles or crossed particles. Particle identification and EDS spectrum generation depend on the SEM image, making brightness and contrast settings critical for distinguishing fibers from the filter substrate. The PA3 software employs an image processing approach. After capturing the SEM image, the brightness and contrast settings are adjusted to ensure that only particles on the filter paper appear bright against a dark background (representing the filter). The software then converts the image into a binary format, identifying particles with an aspect ratio of three (for this particular application). EDS analysis is automatically conducted on these identified particles matching the set aspect ratio.

However, if the brightness and contrast settings are inadequate, both particles and portions of the filter may become visible in the SEM image. In such cases, parts of the filter may be mistakenly identified as fibers. Nevertheless, while the processing time will increase, since the elemental composition of these misidentified fibers does not match that of zeolite fibers, they will not be mis-categorized.

Furthermore, the method cannot detect fibers smaller than a pixel in the chosen image resolution. For example, at 2000X magnification and an image resolution of 2048× 1536 pixels, the identification of fibers smaller than 0.5 mm can be problematic. Although manual counting is highly labor-intensive and impractical for large-scale environmental sampling, it can be used to identify fibers that are crossed or attached to other particles, with manual EDS analysis able to be performed to confirm their composition (Alexandrov et al., 2015).

## 4. Conclusions

This study presents a novel methodology for the automated detection and quantification of airborne mineral fibers using a desktop SEM integrated with EDS analysis and particle analysis. The results from the performance evaluation study demonstrate a strong linear relationship (R^2^ = 0.98) between the erionite mass percentage in bulk samples and fiber concentrations in aerosolized air, with fiber counts ranging from 0 f/cc to 716 f/cc. The detection limit of 7.4 f/cc indicates that this automated method can achieve high sensitivity. However, further assessment is required for the detection of fibers at lower erionite concentrations. The user has the flexibility to decide on the number of FOVs to be analyzed based on the application, the analytical sensitivity required, and the time available for analyzing each sample.

This automated approach addresses many limitations associated with manual fiber counting techniques, such as labor intensity, operator bias, and long processing times. By utilizing a motorized SEM stage and advanced particle analysis software, the methodology enables fiber detection over large areas of polycarbonate filters, significantly enhancing throughput and analytical sensitivity. The application of the method to both performance evaluation (PE) standards and real-world samples from Auckland, New Zealand demonstrates its usefulness in detecting fibers in complex environmental matrices, including dust and other particulate matter.

However, while the method shows great promise, several challenges remain. The preparation of performance evaluation standards at extremely low concentrations is difficult, and the accuracy of the method at lower fiber count concentrations needs further validation. Additionally, limitations such as electron beam drift and the difficulty in distinguishing crossed or attached fibers during automated analysis highlight areas for improvement. Despite these limitations, the automated process significantly reduces human intervention, enhances efficiency, and ensures consistency in fiber detection, making it a valuable addition to current monitoring technologies.

Furthermore, integrating this approach with other analytical techniques, such as TEM, could provide further insights into the nature and composition of airborne mineral fibers, aiding in more comprehensive environmental risk assessments. This work lays the foundation for scalable, high-throughput analysis of airborne fibers, contributing to better public health and environmental safety practices.

## Supplementary Material

Supporting Information final 250803

Appendix A. Supplementary data

Supplementary data to this article can be found online at https://doi.org/10.1016/j.atmosenv.2025.121217.

## Data Availability

Data will be made available on request.

## HIGHLIGHTS

- A novel automated detection and quantification method for elongated airborne mineral particles presented.
- Fiber concentration validations demonstrate a strong linear correlation (R^2^ = 0.98).
- The automated method is validated for fibers detection in complex environmental matrices from Auckland.
- Provides a high-throughput analytical framework for airborne mineral fiber identification.

## List of abbreviations

EMPs: Elongated Mineral Particles
PCM: Phase Contrast Microscopy
SEM: Scanning Electron Microscopy
EDS: Energy Dispersive Spectroscopy
PC: Polycarbonate
PE: Performance evaluation
NOA: Naturally Occurring Asbestos
TEM: Transmission Electron Microscopy
XRPD: X-ray powder diffraction
EPMA: Electron probe microanalysis
FOVs: Fields of Views
ISO: International Standard Organization
NIOSH: National Institute for Occupational Safety and Health
SEM-EDS: Scanning electron microscopy energy-dispersive X-ray spectrometry
XRD: X-Ray diffraction
Si: Silica
Al: Aluminum
Na: Sodium
K: Potassium
Ca: Calcium
Mg: Magnesium
Cl: Chlorine
