# Supplementary material for: A novel approach for quantifying elongated airborne mineral particles (EMPs) using an automated scanning electron microscope (SEM)
Source: Atmos Environ (1994). Author manuscript; Available in PMC 2026 Jan 29. (PMC12851432; doi:10.1016/j.atmosenv.2025.121217)
Supplement: Supporting Information final 250803 [file NIHMS2100866-supplement-Supporting_Information_final_250803.pdf]

A Novel Approach for Quantifying Elongated Airborne Mineral Particles (EMPs) Using an Automated Scanning Electron Microscope (SEM)

Anushka Elangasinghe [a], Hamesh Patel [a,b,c], Kim N. Dirks [d], Ayrton Hamilton [a], Wenxia (Wendy) Fan [a], Shuoyu Chen [d], Nick Talbot [e], Shanon Lim [a,f], Jed Januch[g], Martin Brook [a], Brett Wells [b], David E. Williams [b,h], Perry Davy[i], Woodrow Pattinson[j], Jennifer A. Salmond [a]

[a] School of Environment, Faculty of Science, University of Auckland, Private Bag 92019, Auckland, New Zealand

[b] Mote Limited, 40a George Street, Mount Eden, Auckland, New Zealand

[c] International Laboratory for Air Quality & Health (ILAQH), Queensland University of Technology, 2 George Street, Brisbane, Queensland, 4000, Australia

[d] Department of Civil and Environment Engineering, Faculty of Engineering, University of Auckland, Auckland, New Zealand

[e] Environment Southland, Cnr North Rd &, Price Street, Waikiwi, Invercargill, New Zealand

[f] Pattle Delamore Partners, Level 2/109 Fanshawe Street, Auckland 1010, New Zealand

[g] U.S. Environmental Protection Agency, Region 10, 7411 Beach Drive East, Port Orchard, WA 98336

[h] School of Chemical Sciences, Faculty of Science, University of Auckland, Private Bag 92019, Auckland, New Zealand

[i] Institute of Geological and Nuclear Sciences, Wellington, New Zealand

[j] Deceased 15 March 2020

Supporting Information

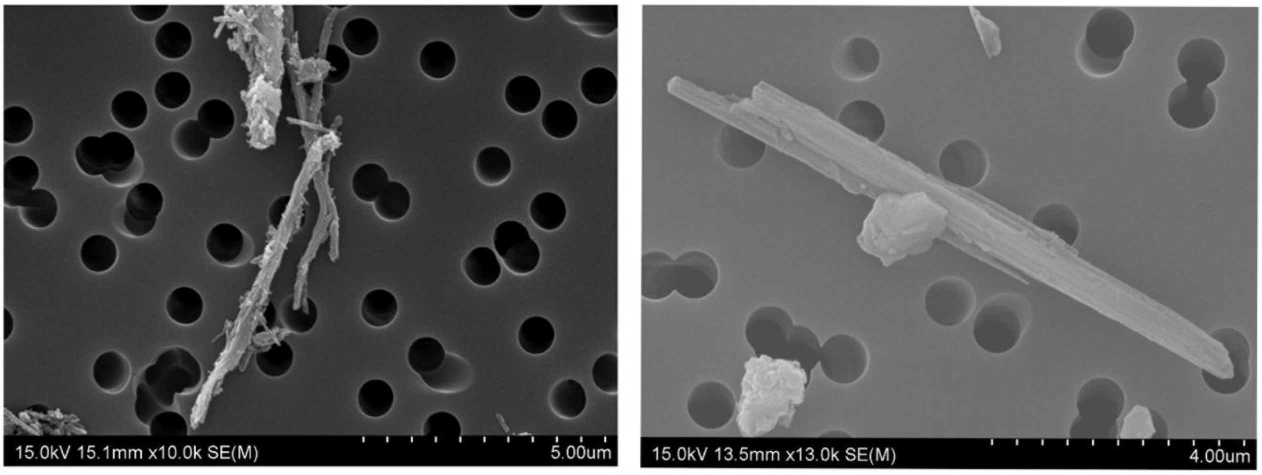

(a) Water-spiked erionite (b) Air-spiked erionite

Fig. S1: Comparison of water-spiked and air-spiked erionite on the PC filter

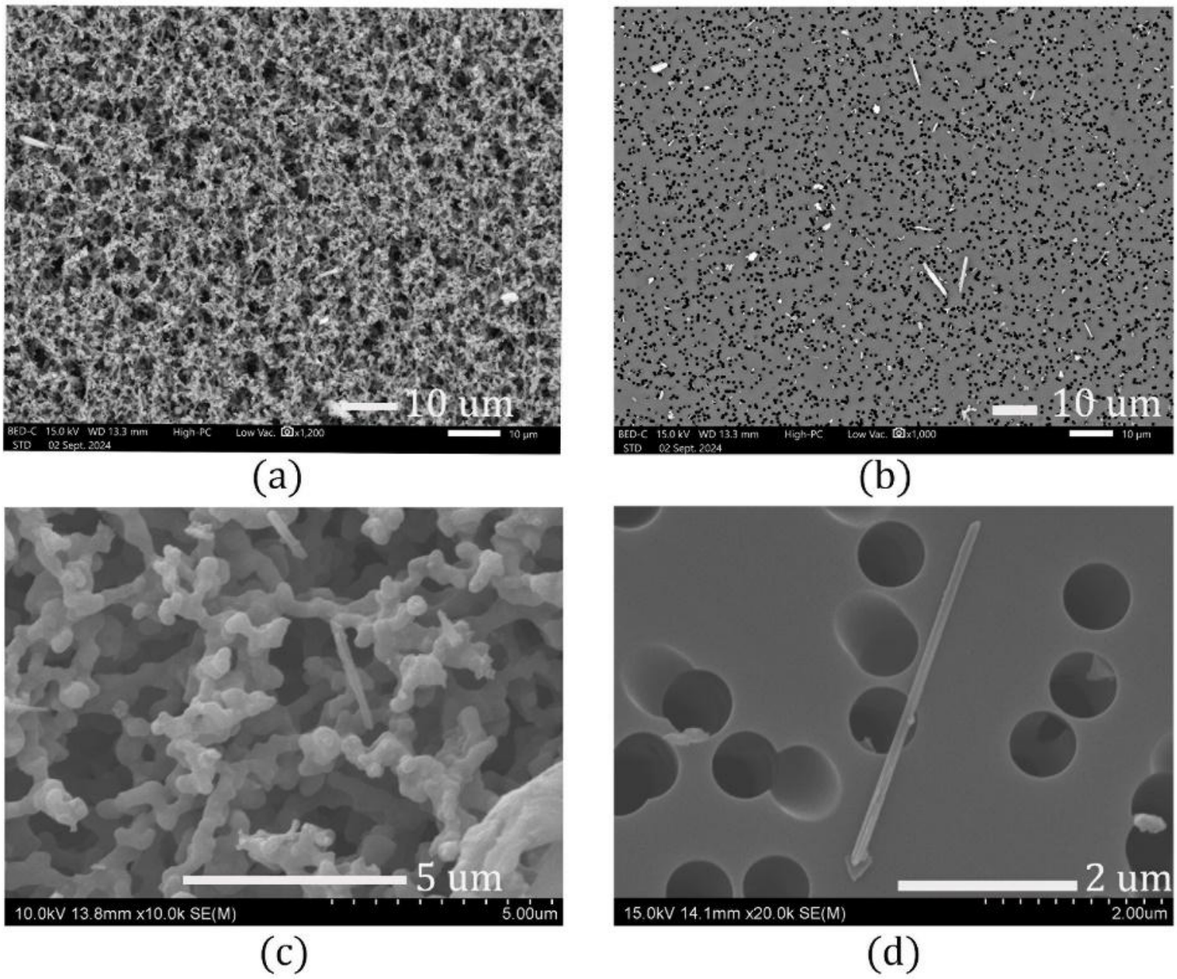

Fig. S2: Comparison of spiked-erionite on MCE and PC filters (a) and (b), The same amount of erionite spiked on MCE and PC filters respectively. (c) A higher magnification image of the MCE filter showing fine fibres hidden among the structure of the MCE filter (d) A higher magnification image of the PC filter holding the fibre on top of the filter

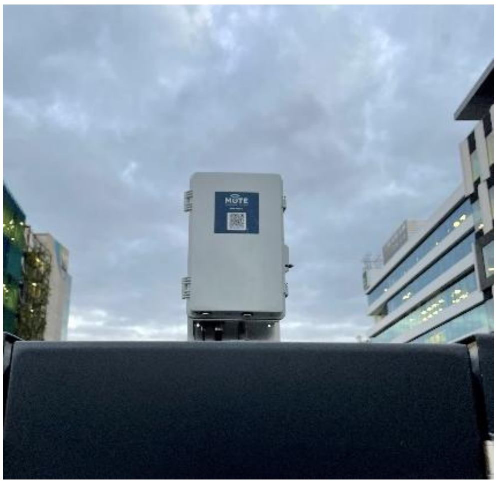

Fig. S3: Image of the FilterMote sampling device.

Table S1: Details of real-world sampling locations

The site code	Sampling Location	Average sample flow rate (l/min)	Sampling time (min)	Sampling Volume (m^3)
ER 6	Riverhead (static)	3.36	10079	33.8
ER 11	Riverhead (static)	3.40	10079	34.3
ER 12	Riverhead (static)	3.23	10079	32.6
ER 13	Riverhead (static)	3.64	10079	36.7
ER 16	Riverhead (static)	3.24	10079	32.7
A-B	Te Henga (Quarry inner road)	3.0	29	0.09

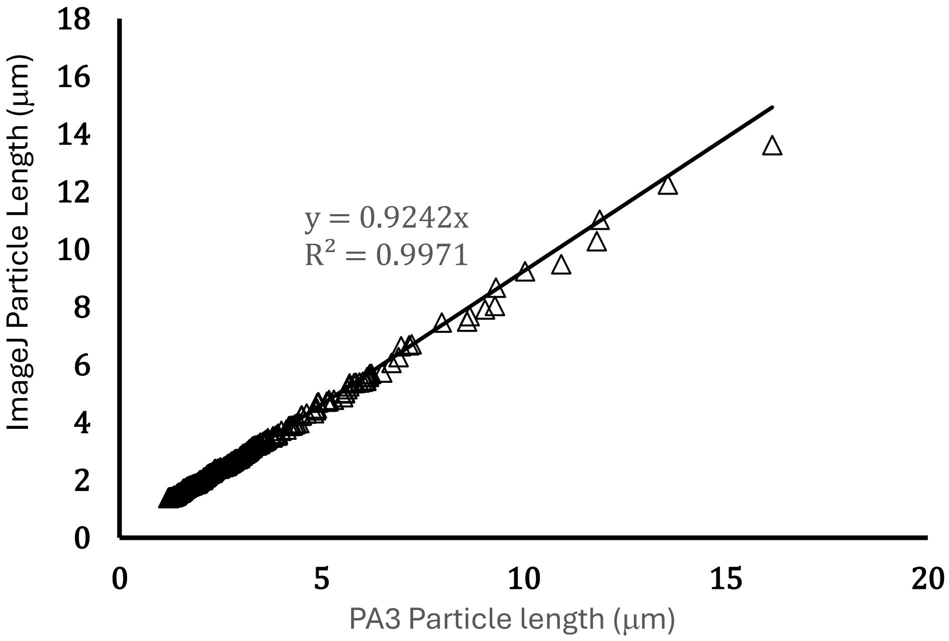

Fig. S4: Length of a particle obtained by PA3 against the length estimates obtained by ImageJ software

Table S2: Results obtained by automated counting of erionite fibers on PE standard filters

Sample number	Erionite in the bulk sample (% mass)	Erionite fiber count on SEM images at 2000X over 25 FOVs								
		Transect 1	Transect 2	Transect 3	Transect 4	Transect 5	Total	f/cc	95% Confidence interval	
									Upper limit f/cc	Lower limit f/cc
PC1	0	0	0	0	0	0	0	0	0	7.4
PC2	0.02	1	2	1	0	1	5	12.4	5.0	29.7
PC3	0.15	4	2	6	8	4	26	64.4	42.1	94.1
PC4	0.37	10	8	3	11	5	36	89.1	61.9	123.8
PC5	0.89	19	16	21	23	26	105	260.0	200.5	302.0
PC6	1.74	49	63	57	42	78	289	715.5	638.8	799.7

Table S3: Approximate analysis time for different numbers of FOV of a sample SEM image at 2000X using the automated method

Number of FOV	25	50	75	100	200	300	400
Time/ (hours)	3±1	7±2	11±3	15±5	30±8	44±10	60±15

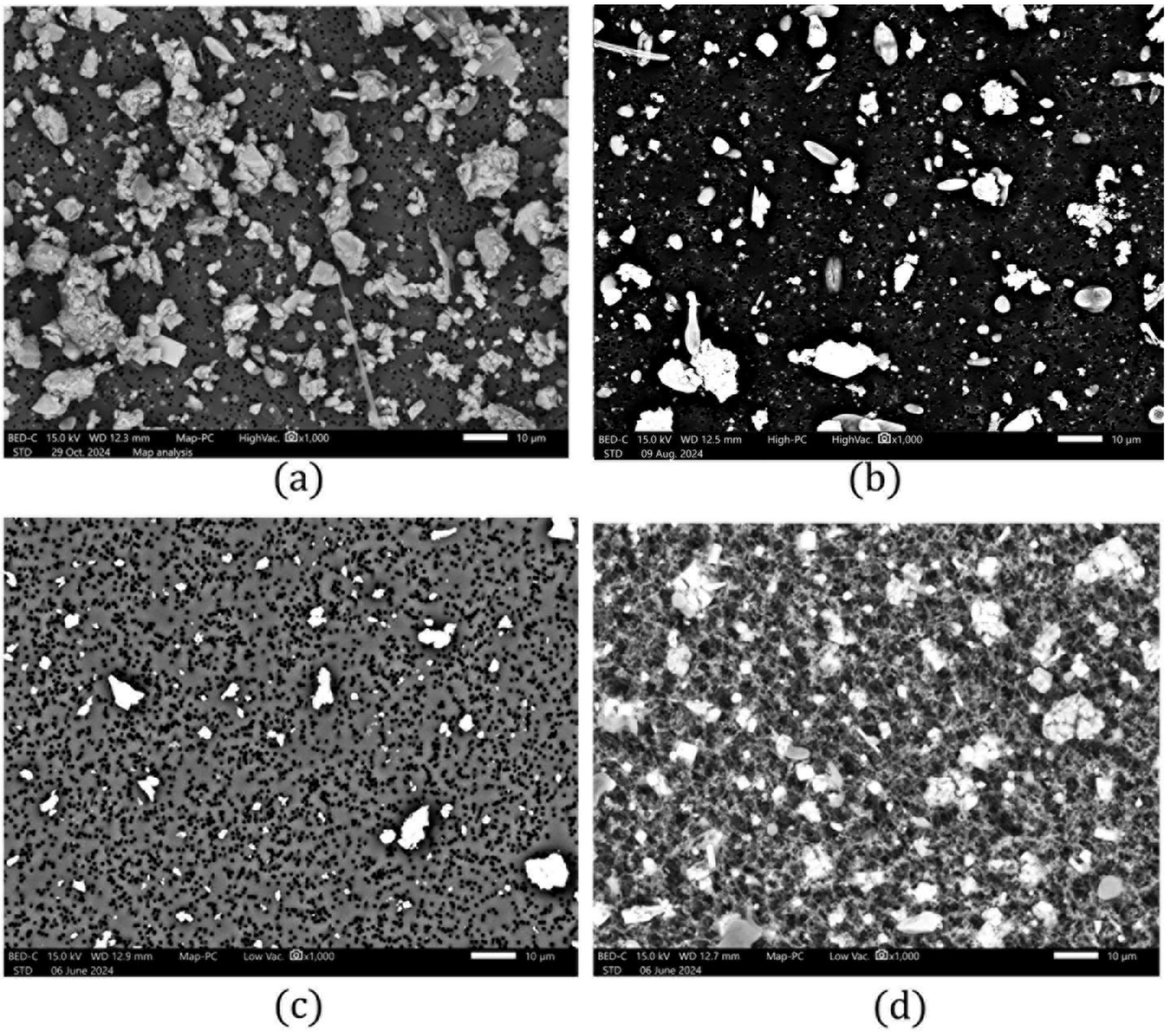

Fig. S5: Different filter loadings of real-world samples: (a) An overloaded PC filter, unsuitable for automated fiber counting due to particle overlapping (sampling time: one week at 3.3 L/min). (b) A moderately loaded PC filter from ambient sampling, with brightness and contrast adjusted, suitable for automated counting (sampling time: one week at 3.3 L/min). (c) A moderately loaded filter near a gravel road, showing good contrast between the particles and the filter substrate (sampling time: 30 min at 3.3 L/min). (d) An MCE filter sample for comparison, where some particles are hidden within the spongy structure of the filter (sampling time: one week at 3.3 L/min).

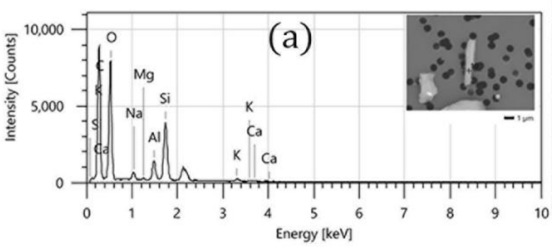

Items	Value
measurement conditions	
Acceleration voltage	15.00 kV
Probe current	-
Magnification	x 10000
Process time	T2
Measurement detector	First
Live time	10.00 seconds
Real time	11.64 seconds
Dead time	13.00 %
Count rate	23562.00 CPS

Element	10s Mass%	Atom%
C	49.55 ± 0.09	58.65 ± 0.11
O	41.14 ± 0.15	36.56 ± 0.13
Na	1.03 ± 0.02	0.64 ± 0.01
Mg	0.13 ± 0.01	0.08 ± 0.01
Al	1.84 ± 0.02	0.97 ± 0.01
Si	5.70 ± 0.04	2.89 ± 0.02
K	0.50 ± 0.02	0.18 ± 0.01
Ca	0.12 ± 0.01	0.04 ± 0.01

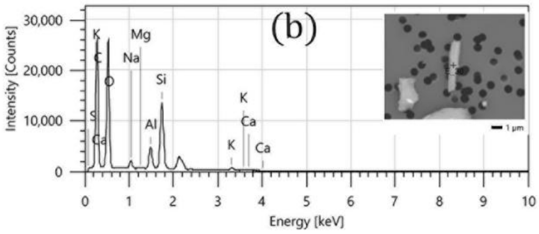

Items	Value
measurement conditions	
Acceleration voltage	15.00 kV
Probe current	-
Magnification	x 10000
Process time	T2
Measurement detector	First
Live time	30.00 seconds
Real time	35.19 seconds
Dead time	14.00 %
Count rate	26068.00 CPS

Element	30s Mass%	Atom%
C	48.32 ± 0.05	57.56 ± 0.06
O	41.66 ± 0.08	37.26 ± 0.08
Na	0.99 ± 0.01	0.61 ± 0.01
Mg	0.10 ± 0.01	0.06 ± 0.00
Al	2.04 ± 0.01	1.08 ± 0.01
Si	6.31 ± 0.02	3.21 ± 0.01
K	0.47 ± 0.01	0.17 ± 0.00
Ca	0.12 ± 0.01	0.04 ± 0.00

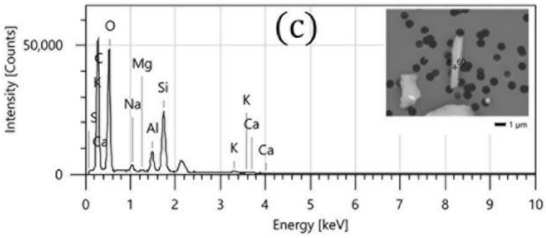

Items	Value
measurement conditions	
Acceleration voltage	15.00 kV
Probe current	-
Magnification	x 10000
Process time	T2
Measurement detector	First
Live time	60.00 seconds
Real time	69.71 seconds
Dead time	13.00 %
Count rate	24590.00 CPS

Element	60s Mass%	Atom%
C	49.25 ± 0.04	58.34 ± 0.05
O	41.47 ± 0.06	36.88 ± 0.05
Na	0.85 ± 0.01	0.53 ± 0.01
Mg	0.12 ± 0.00	0.07 ± 0.00
Al	1.94 ± 0.01	1.02 ± 0.01
Si	5.86 ± 0.02	2.97 ± 0.01
K	0.40 ± 0.01	0.14 ± 0.00
Ca	0.12 ± 0.01	0.04 ± 0.00

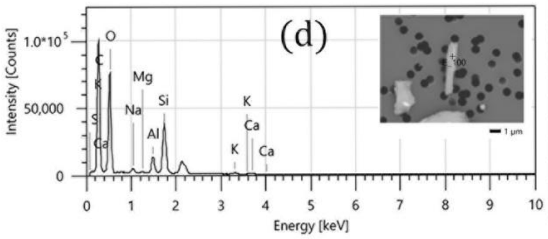

Items	Value
measurement conditions	
Acceleration voltage	15.00 kV
Probe current	-
Magnification	x 10000
Process time	T2
Measurement detector	First
Live time	100.00 seconds
Real time	116.69 seconds
Dead time	14.00 %
Count rate	25425.00 CPS

Element	100s Mass%	Atom%
C	52.03 ± 0.03	60.89 ± 0.03
O	39.79 ± 0.05	34.97 ± 0.04
Na	0.62 ± 0.01	0.38 ± 0.00
Mg	0.09 ± 0.00	0.05 ± 0.00
Al	1.75 ± 0.01	0.91 ± 0.00
Si	5.29 ± 0.01	2.65 ± 0.01
K	0.32 ± 0.00	0.12 ± 0.00
Ca	0.12 ± 0.00	0.04 ± 0.00

Fig. S6: EDS spectra of an erionite fiber obtained at different analysis times, showing the identification of all elements with an increase in intensity counts at longer analysis times: (a) 10 s, (b) 30 s, (c) 60 s, and (d) 100 s.

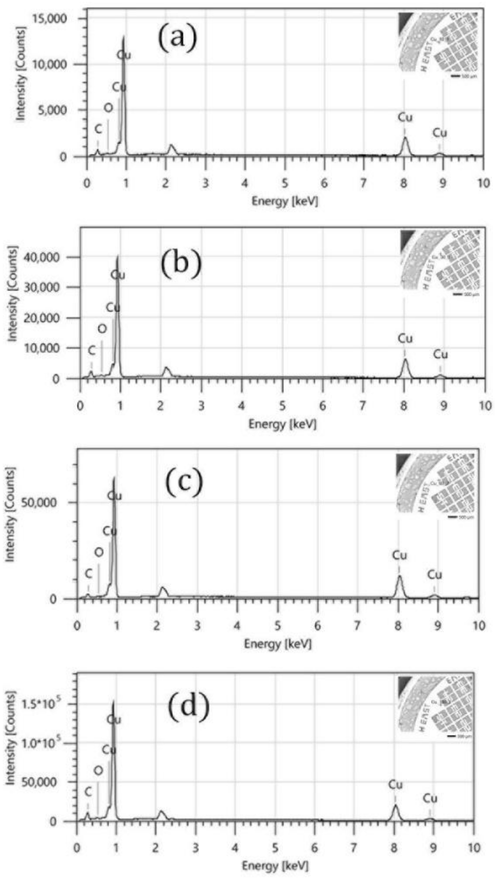

Items	Value
measurement conditions	
Acceleration voltage	15.00 kV
Probe current	-
Magnification	x 30
Process time	T2
Measurement detector	First
Live time	10.00 seconds
Real time	12.23 seconds
Dead time	17.00 %
Count rate	31002.00 CPS

10s		
**Element**	**Mass%**	**Atom%**
C	8.11 ± 0.07	31.45 ± 0.26
O	0.57 ± 0.02	1.66 ± 0.07
Cu	91.32 ± 0.57	66.89 ± 0.42

Items	Value
measurement conditions	
Acceleration voltage	15.00 kV
Probe current	-
Magnification	x 30
Process time	T2
Measurement detector	First
Live time	30.00 seconds
Real time	36.74 seconds
Dead time	17.00 %
Count rate	30885.00 CPS

30s		
**Element**	**Mass%**	**Atom%**
C	9.69 ± 0.04	35.82 ± 0.15
O	0.52 ± 0.01	1.45 ± 0.04
Cu	89.78 ± 0.32	62.73 ± 0.23

Items	Value
measurement conditions	
Acceleration voltage	15.00 kV
Probe current	-
Magnification	x 30
Process time	T2
Measurement detector	First
Live time	60.00 seconds
Real time	71.71 seconds
Dead time	16.00 %
Count rate	26127.00 CPS

60s		
**Element**	**Mass%**	**Atom%**
C	9.22 ± 0.03	34.62 ± 0.11
O	0.46 ± 0.01	1.30 ± 0.03
Cu	90.32 ± 0.23	64.08 ± 0.17

Items	Value
measurement conditions	
Acceleration voltage	15.00 kV
Probe current	-
Magnification	x 30
Process time	T2
Measurement detector	[illegible]
Live time	100.00 seconds
Real time	124.05 seconds
Dead time	19.00 %
Count rate	33790.00 CPS

10s		
**Element**	**Mass%**	**Atom%**
C	13.22 ± 0.03	44.09 ± 0.09
O	0.64 ± 0.01	1.61 ± 0.02
Cu	86.14 ± 0.17	54.30 ± 0.11

Fig. S7: EDS spectra of copper obtained at different analysis times, showing the identification of copper, along with carbon and oxygen (originating from the filter substrate). An increase in intensity counts is observed with longer analysis times: (a) 10 s, (b) 30 s, (c) 60 s, and (d) 100 s.

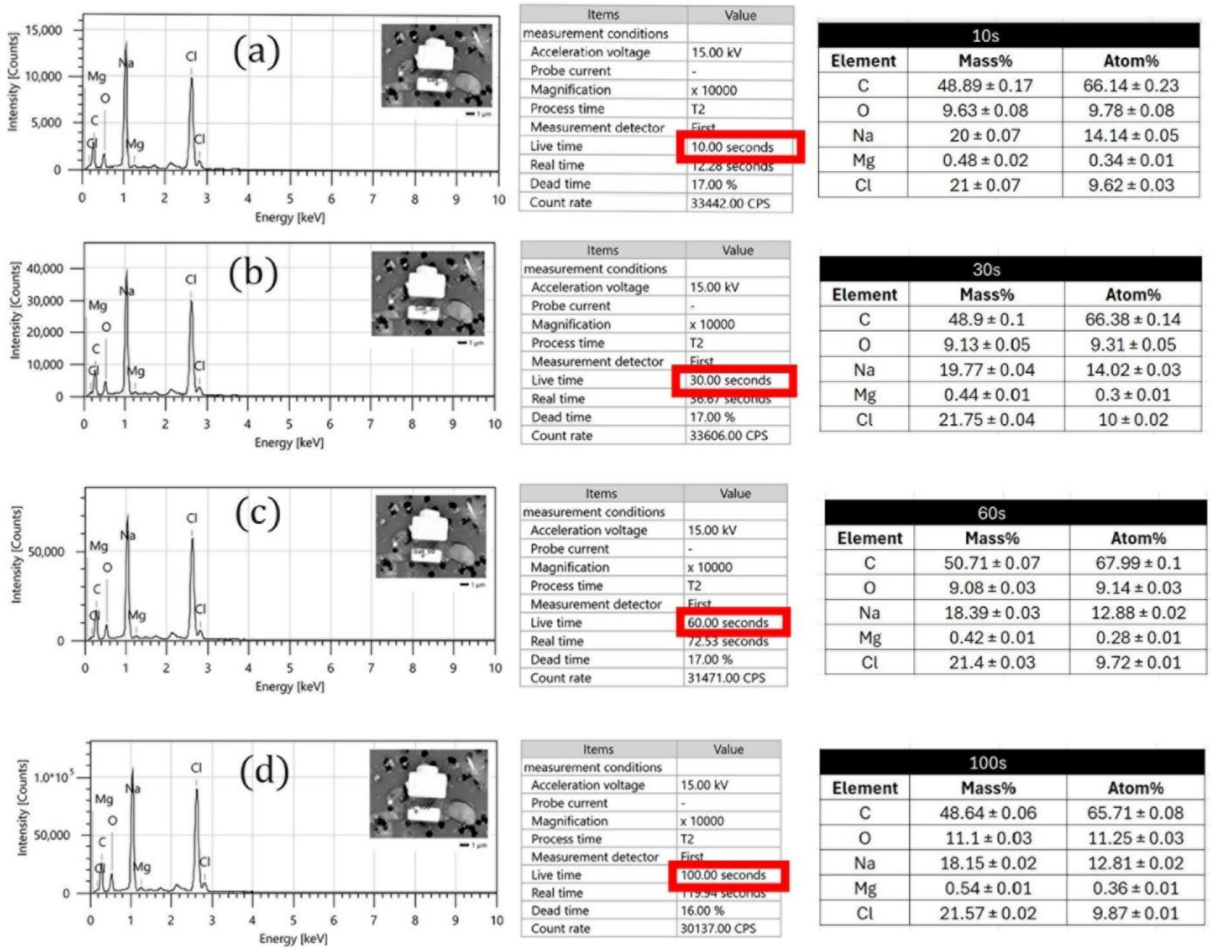

Fig. S8: EDS spectra of a salt crystal obtained at different analysis times, showing the identification of sodium, chlorine, and trace magnesium, along with carbon and oxygen (originating from the filter substrate). An increase in intensity counts is observed with longer analysis times: (a) 10 s, (b) 30 s, (c) 60 s, and (d) 100 s.

Extract X

Extracted from ISO 14966:2019 (ISO - International Organization for Standardization, 2019b)

7.4.2.7 Termination of fibre counting

Fibre counting can be terminated early with respect to a fibre type as a function of a limit or guide value K_R (fibres per m^3). If more than N_A fibres of this type have been found counting can be terminated. N_A is calculated as follows:

$$N_A = \frac{3. K_R. \acute{v}_S}{F_A}$$

N_A is the fibre number of a specific type

K_R is the benchmark or limit to be tested, in m^{-3}

$\acute{v}_S$ is dependent from v_S the sampled volume of air per filter area

$\acute{v}_S$ = 1m³/cm³ for $v_S \leq$ 1 m³/cm²

$\acute{v}_S$ = 1m³/cm³ for v_S > 1 m³/cm²

F_A is a constant (=100 cm^{-2})

In cases where limit or guide values do not exist, the stop criterion can be freely defined for each individual measurement in accordance with the specific measurement objective. Here it is essential that on account of the associated measurement uncertainty N_A is not set too low. N_A shall be at least 15. The analysed filter area shall not be less than 0.25 mm^2.
